# NSs of the mildly virulent sandfly fever Sicilian virus is unable to inhibit interferon signaling and upregulation of interferon-stimulated genes

**DOI:** 10.1099/jgv.0.001676

**Published:** 2021-11-02

**Authors:** Jennifer Deborah Wuerth, Friedemann Weber

**Affiliations:** ^1^​ Institute for Virology, FB10-Veterinary Medicine, Justus-Liebig University, Giessen, Germany; ^2^​ Institute of Innate Immunity, University of Bonn, Bonn, Germany

**Keywords:** interferon antagonism, interferon-stimulated gene, interferon-regulatory factor, NSs protein, phlebovirus, sandfly fever Sicilian virus

## Abstract

Phleboviruses (order *Bunyavirales*, family *Phenuiviridae*) are globally emerging arboviruses with a wide spectrum of virulence. Sandfly fever Sicilian virus (SFSV) is one of the most ubiquitous members of the genus *Phlebovirus* and associated with a self-limited, incapacitating febrile disease in travellers and military troops. The phleboviral NSs protein is an established virulence factor, acting as antagonist of the antiviral interferon (IFN) system. Consistently, we previously reported that SFSV NSs targets the induction of IFN mRNA synthesis by specifically binding to the DNA-binding domain of the IFN transcription factor IRF3. Here, we further characterized the effect of SFSV and its NSs towards IFN induction, and evaluated its potential to affect the downstream IFN-stimulated signalling and the subsequent transactivation of antiviral interferon-stimulated genes (ISGs). We found that SFSV dampened, but did not entirely abolish type I and type III IFN induction. Furthermore, SFSV NSs did not affect IFN signalling, resulting in substantial ISG expression in infected cells. Hence, although SFSV targets IRF3 to reduce IFN induction, it is not capable of entirely disarming the IFN system in the presence of high basal IRF3 and/or IRF7 levels, and we speculate that this significantly contributes to its low level of virulence.

## Introduction

Phleboviruses (order *Bunyavirales*, family *Phenuiviridae,* genus *Phlebovirus*) are gobally emerging arboviruses that cover a broad range of virulence [1–3]. The disease spectrum among well-known members ranges from seasonal, self-limited febrile disease (sandfly fever Sicilian virus (SFSV), Punta Toro virus (PTV)), via fever complicated by meningitis and encephalitis (Toscana virus, TOSV) to acute hepatitis, encephalitis, ocular complications, or haemorrhagic fever (Rift Valley fever virus, RVFV) [1–3]. While some novel virulent members were isolated from clinically apparent patients [4, 5], the majority of novel phleboviruses is curently identified by vector screening and subsequent sequence analysis, leaving their disease potential only partially explored or entirely elusive [6–10].

Historically, an outbreak of an incapacitating febrile disease accompanied by sudden onset generalized myalgia, headaches, malaise, ocular and gastrointestinal symptoms, termed *‘sandfly fever’*, *‘3 day fever’*, *‘pappataci fever’*, or *‘dog disease’* during the Sicilian invasion of World War II led to the first isolation of SFSV from infected soldiers [11, 12]. SFSV is also one of the most widespread phlebovirus, with its endemic area ranging from Portugal across the Mediterranean basin to as far east as Bangladesh, and south to Somalia, and seroprevalence levels reaching up to 50 % in humans and around 80 % in domestic animals [5, 13–17]. Consequently, SFSV continues to cause disease in immunologically naïve groups such as deployed military personnel and travellers [18–22]. Furthermore, several SFSV-like viruses were recently identified, such as sandfly fever Turkey, Dashli, Toros and Zerdali viruses [4, 23, 24].

Phleboviruses contain a tri-segmented, mainly negative-sense single-stranded RNA genome. While the large (L) and the medium (M) segment encode the viral RNA-dependent RNA polymerase and the glycoproteins, respectively, the small (S) segment codes for the nucleocapsid protein N that packages the genomic and antigenomic RNA into viral nucleoprotein complexes [2]. Additionally, the M segment encodes the non-structural protein NSm and the 78 kDa protein, whereas the S segment contains the gene for the NSs protein in an ambisense orientation. Phlebovirus replication takes place exclusively in the cytoplasm of the host cell, where the viral RNA is sensed by pattern-recognition receptor retinoic-acid inducible gene I (RIG-I) [25]. Upon activation, RIG-I engages the adapter, mitochondrial antiviral signalling protein (MAVS), providing a platform for the phosphorylation of interferon-regulatory factors IRF3 and IRF7 and culminating in the induction of type I and type III interferons (IFN-α/β and IFN-λ, respectively) [26, 27]. Secreted IFNs then stimulate their cognate receptors in a para- and autocrine manner, which in turn mediates the phosphorylation of the signal transducers and activators of transcription 1 (STAT1) and STAT2. A complex of STAT1, STAT2 and IRF9, so-called IFN-stimulated gene factor 3 (ISGF3), transactivates an assortment of interferon-stimulated genes (ISGs) to establish an antiviral state in the cell [28]. The dynamin-like GTPase MxA and ubiquitin-like protein ISG15 are examples of ISGs with anti-phleboviral activity [29–31] and the protective effect of the IFN response has been illustrated for several phleboviruses, including SFSV [29, 32–41]. With NSs, however, phleboviruses express an IFN antagonist that can display a multitude of strategies for curbing IFN induction [3, 42, 43]. Depending on the virus, IFN antagonisms range from a global, general block of host-gene expression to fine-adjusted targeting of specific host factors. Mechanistically, some NSs proteins are driving proteasomal degradation of target host factors, whereas others engage in their stoichiometric binding and sequestration [34, 44–48]. For SFSV NSs, we previously reported that it acts as suppressor of type IFN induction by obstructing the DNA-binding domain of the IFN transcription factor IRF3 [47]. Thus, SFSV employs a specific rather than a global or destructive strategy as used by other, more virulent phleboviruses. Here, given its stoichiometric and highly IRF3-specific mechanism of action, we evaluated the efficiency and breadth by which SFSV is counteracting the induction of different types of IFNs, and extended our analyses to downstream events like IFN signalling and ISG expression. Our results indicate that SFSV NSs is a modulator rather than a strong antagonist of IFN induction that is exclusively acting on IRF3, implying a possible correlation between the strength of a particular phleboviral NSs protein and the associated virulence.

## Methods

### Cells, viruses, infection, and plasmids

A549, BHK-21, HEK293, HepG2, Vero B4 and Vero E6 cells were maintained in CCM34 medium (DMEM with addition of 17.8 mg l^−1^ l-alanine, 0.7 g l^−1^ glycine, 75 mg l^−1^ l-glutamic acid, 25 mg l^−1^ l-proline, 0.1 mg l^−1^ biotin, 25 mg l^−1^ hypoxanthine, and 3.7 g l^−1^ sodium bicarbonate) supplemented with 10 % FCS, 2 mM glutamine, 100 U ml^−1^ penicillin, and 100 µg ml^−1^ streptomycin.

The Sabin strain of SFSV was propagated in Vero B4 cells, attenuated RVFV strains MP12 and clone 13 in BHK-21 cells. Virus titres were determined via plaque assay on Vero E6 cells with Avicel overlay and crystal violet staining. Cell lines and virus stocks were routinely tested for mycoplasma contamination, and virus stocks were tested for the presence of defective interfering particles. For infection, A549 cells (1×10^5^ per 24-well) were washed with sterile PBS and inoculated with virus diluted to the respective multiplicity of infection (MOI) in serum-free medium for 1 h at 37 °C, after which the inoculate was replaced with fully supplemented medium. For super-stimulation or inhibition of IFN signalling, cells were treated with 100 U ml^−1^ of pan-species IFN-α (B/D) (PBL Assay Science) [49], IFN-β (Betaferon, Schering) or ruxolitinib (INCB018424, Selleckchem), respectively, from 1 h prior to infection until cell lysis.

Expression constructs encoding 3×FLAG-tagged NSs of SFSV (GenBank EF201822.1), pI.18-NSsRVFV-3×FLAG and pI.18–3×FLAG-ΔMx were described before [45, 47]. Expression constructs for the NSs proteins of TOSV prototype strain ISS.Phl.3 and SFTSV strain HB29 (GenBank X53794.1 and NC_018137.1, respectively) were synthesized (BioCat and Eurofins Genomics) and subcloned into pI.18. Luciferase reporter constructs p-125Luc [50] and pGL3-Mx1P-Luc [51] were kindly provided by Takashi Fujita and Georg Kochs, respectively. pRL-SV40 was purchased from Promega.

### Immunoblot analysis

Samples were run on 12 % acrylamide gels using the Tris-glycine buffer system and transferred to polyvinylidene fluoride (PVDF) membranes (Millipore) via semidry blotting. Membranes were blocked in TBS containing 5 % BSA or milk powder, stained with primary antibody for 1 h at room temperature or overnight at 4 °C, washed in TBS/0.1 % Tween-20, stained with secondary antibodies for 45 min, washed again in TBS/0.1 % Tween-20 and once in TBS, and finally developed with SuperSignal West Femto kit (Pierce). Bands were detected using a ChemiDoc Imaging System (Bio-Rad) or a Sapphire Biomolecular Imager (Azure Biosystems).

Primary antibodies comprised: ISG15 (ab36765, Abcam, 1 : 500), MxA (Sigma, MABF938, 1 : 1000), RIG-I (ag-20b-0009, AdipoGen, 1 : 1000), RVFV N (rabbit hyperimmune serum, provided by Alenjandro Brun, 1 : 1000), SFSV N (mouse immune ascites fluid, provided by WRCEVA, 1 : 1000), p-STAT1(Y701) (7649, Cell Signalling, 1 : 1000), STAT1 (610186, BD Transduction Laboratories, 1 : 1000), p-STAT2(Y690) (88410, Cell Signalling, 1 : 1000), STAT2 (610188, BD Transduction Laboratories, 1 : 1000), tubulin (ab6046, Abcam, 1 : 2500). Secondary antibodies were anti-mouse (0031430 1892913) and anti-rabbit (0031460 1892914, both Thermo Fisher).

### Human IFN-λ1/3 ELISA

Supernatants of infected A549 cells were diluted 1 : 5 in medium and subjected to human IFN-lambda 1/3 DuoSet ELISA (DY1598B, R and D Systems) according to the manufacturer’s specifications.

### Reverse transcriptase (RT)-PCR

RNA was isolated using the RNeasy Mini Kit (Qiagen) and then subjected to DNase I digest and cDNA synthesis using PrimeScript RT reagent Kit with gDNA Eraser (TaKaRa) as recommended by the manufacturers. Host transcripts were detected with SYBR Premix Ex Taq (Tli RNaseH Plus) (TaKaRa) and QuantiTect primers (*DDX58:* QT00040509; *IFNB1*: QT00203763; *IFNL1*: QT01033564; *IFNL2/3*: QT00222488; *IRF3:* QT00012866; *IRF5*: QT00210595, *IRF7*: QT00210595; *ISG15*: QT00072814; *MX1*: QT00090895; *RRN18S*: QT00199367, Qiagen). Premix Ex Taq (Probe qPCR) (TaKaRa) was used to detect viral RNA with previously published primers and probes for the SFSV and RVFV L segments (SFSV L: fwd 5′-TCT GAG AAC TGA GCT ACA AGT GTT TAT TA-3′, rev 5′-TTC CCA TCT CTC TTC TGA AGA GTG-3′, probe 6-FAM-AGG TCA TAG ACA GTA TCA TGA GAA TTG CTA GGT G-BHQ-1 [4]; SFSV S, fwd, 5′-TGC ACT CAT CCA AGC TAT GTG-3′, rev, 5′-GAG GGC TAC AAA CAA GGG ATC-3′, probe, FAM-TCC CCC ATT CTC AGA ATG TAA GAC ATT AGC-BHQ-1 [52]; RVFV L: fwd 5′-TGA AAA TTC CTG AGA CAC ATG G-3′, rev 5′-ACT TCC TTG CAT CAT CTG ATG-3′, probe 6-FAM-CAA TGT AAG GGG CCT GTG TGG ACT TGT G-BHQ-1 [53]). 18S rRNA was used as housekeeping gene to calculate fold induction according to the ΔΔC_T_ method.

### Dual luciferase assay

HEK293 cells seeded into 96-well plates (1.5×10^4^ per well) were transfected using TransIT-LT1 (Mirus Bio LLC). Transfection mixes included the indicated firefly and *Renilla* luciferase reporter constructs (40 ng each), as well as NSs proteins or the control protein ΔMx (0.1 ng, 1 ng and 10 ng), and were filled up to equal plasmid amounts with empty vector pI.18. For stimulation of IFN induction, an expression plasmid for MAVS was added to the transfection mix (50 ng). Gene expression was allowed for 24 h. Subsequently, cells were either harvested or stimulated with IFN-β or IFN-α B/D (100 U ml^−1^) for 24 h. Cell lysis and determination of luciferase activities were performed using the Dual Luciferase Reporter Assay System (Promega) and a LB 942 TriStar^2^ multimode reader (Berthold Technologies). Firefly luciferase activities were normalized to those of *Renilla* luciferase and the stimulated control samples set to 100 % within each biological replicate. Finally, mean and SD values were calculated across the indicated number of biological replicate datasets.

### siRNA-mediated knockdown and infection

A549 cells (1×10^5^ per 24-well) were subjected to reverse transfection via Lipofectamine RNAiMax (Life Technologies) according to the manufacturer’s recommendations. siRNA (all from Qiagen) comprised control siRNA (1027280), siIRF3 (1027416), siIRF5 (1027416), siIRF7 (1027416) or a pool of four custom-designed siRNA oligonucleotides targeting SFSV NSs (siNSs1 5′-TTG GGT CTT AGT GAT GAG CAT-3′, siNSs2 5′-AAG GGA TCA GCT AAT GTC TTA-3′, siNSs3 5′-TAC AAT AAA TTT CAC ACT CAT-3′, siNSs4 5'- AAG GCT CTT AGC TGG CCA CTA-3′) [47].

## Results

### SFSV infection induces IFN signaling and ISG expression despite inhibition of type I IFN induction

**Fig. 1.**
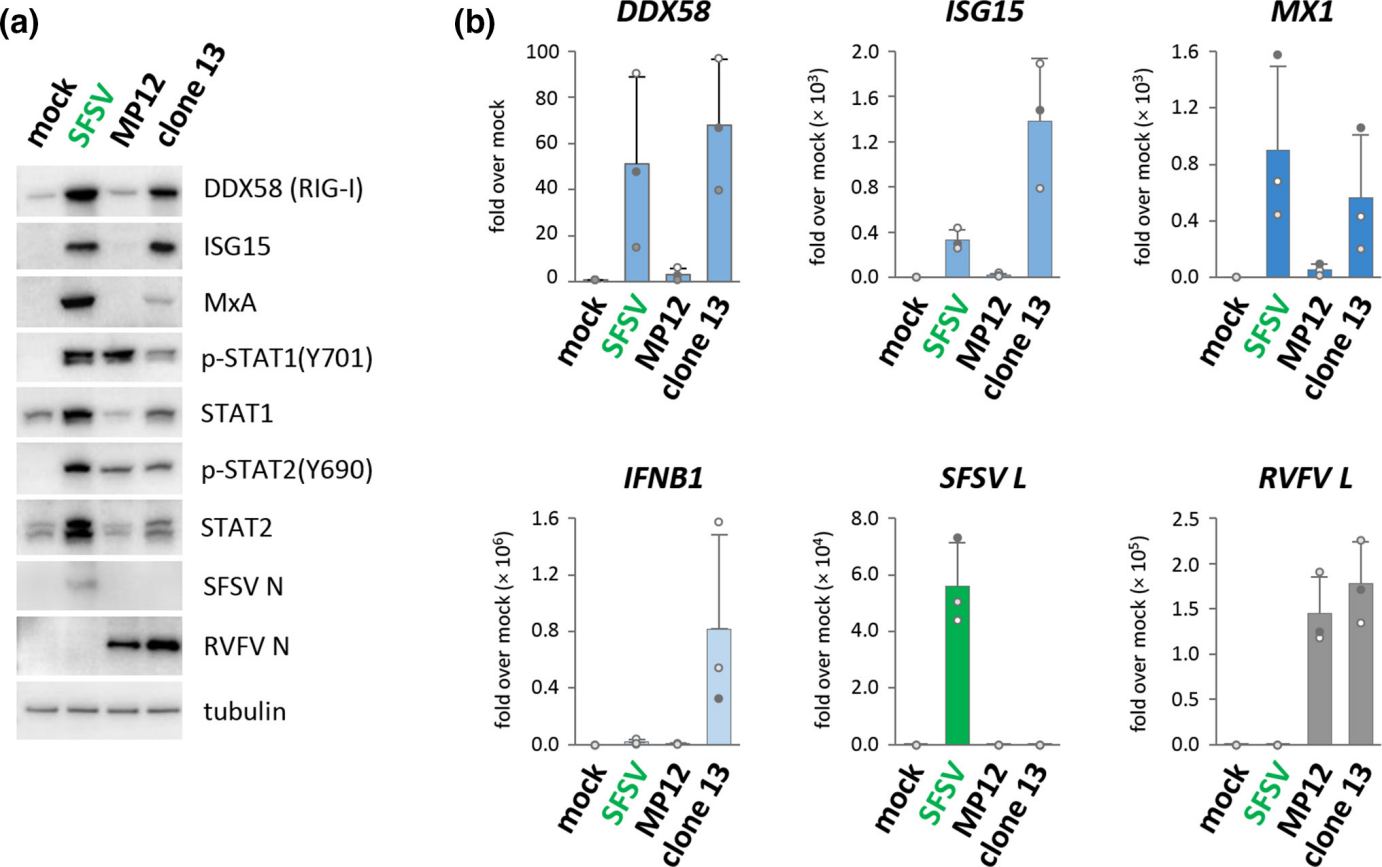
ISG expression and IFN signalling under SFSV infection. (**a**) A549 cells were infected with SFSV, MP12 or clone 13 (MOI 1) and harvested 12 hpi. Lysates were analysed by immunoblot for ISG levels and phosphorylation of STAT1 and STAT2 (*n*=3). (**b**) Matching RNA samples were subjected to quantitative RT-PCR for *IFNB1*, ISGs *DDX58* (encoding RIG-I), *ISG15*, and *MX1*, as well as viral L segments (*n*=3, mean±SD). Please note that, for immunoblotting, antisera with very different signal-to-noise ratio were being used (mouse ascites fluid vs rabbit serum), not permitting any quantitative comparisons between the viral N signals.

SFSV NSs inhibits induction of the *IFNB1* promoter by masking the DNA-binding activity of IRF3 [47]. Nonetheless, the ISG RIG-I is upregulated under infection with parental SFSV or with a recombinant chimeric RVFV expressing SFSV NSs (rZH548ΔNSs::NSsSFSV) [47]. To test whether this was specific to RIG-I or whether ISGs are spared by NSs in general, we interrogated the protein and mRNA levels of two other ISGs, namely ISG15 and MxA [28]. As expected, both these ISGs were strongly upregulated by RVFV strain clone 13, which possesses a large deletion within the NSs gene and is thus a strong IFN and ISG inducer [32], whereas the RVFV strain MP12, harbouring a fully functional NSs, activated them only marginally (Fig. 1a). Infection with SFSV, however, also resulted in elevated levels of RIG-I, MxA, and ISG15 proteins (Fig. 1a). With respect to mRNAs, SFSV infection caused intermediate activation of the *ISG15* gene and strong upregulation of *MX1* and *DDX58* (RIG-I), although only negligible levels of *IFNB1* mRNAs were induced (Fig. 1b). *MX1* is a conserved and strictly IFN-dependent ISG [54]. We therefore assessed the phosphorylation levels of transcription factors STAT1 and STAT2 as proxy for IFN signalling. Indeed, ISG induction under SFSV infection was accompanied by phosphorylation of both STAT1 and STAT2 (Fig. 1a). Additionally, STAT1 (and STAT2), which are also ISGs [28], were elevated on both transcript and protein levels under SFSV infection (Figs 1a and S1a, available in the online version of this article), consistent with their inducibility by IFNs. For STAT1 phosphorylation, we also performed a time course and show that it is detectable already at four hpi and steadily increases until 12 hpi (Fig. S1b).

Of note, pre-treatment with IFN-α prior to infection could not further enhance STAT phosphorylation or ISG expression (fold increase <2, Fig. S2 and data not shown), suggesting that SFSV infection alone already results in maximal ISG induction.

We wondered about the trigger of the IFN signalling and ISG upregulation that occur despite the inhibition of IFN induction by SFSV NSs. One possibility could be the contamination of virus stocks with high amounts of bioactive type III IFNs, as it had been reported previously for hantaviruses [55]. However, inactivation of viral stocks with β-propiolactone, which does not affect IFN bioactivity [56], abolished SFSV replication as expected, but also IFN and ISG induction (Fig. S3 and data not shown). Similarly, when we subjected viral stocks to ultrafiltration, the ISG-inducing activity was retained together with the viral particles by the filter membrane (data not shown). Thus, SFSV itself appears to stimulate the observed IFN signalling.

### SFSV NSs does not affect IFN signaling or ISG induction

**Fig. 2.**
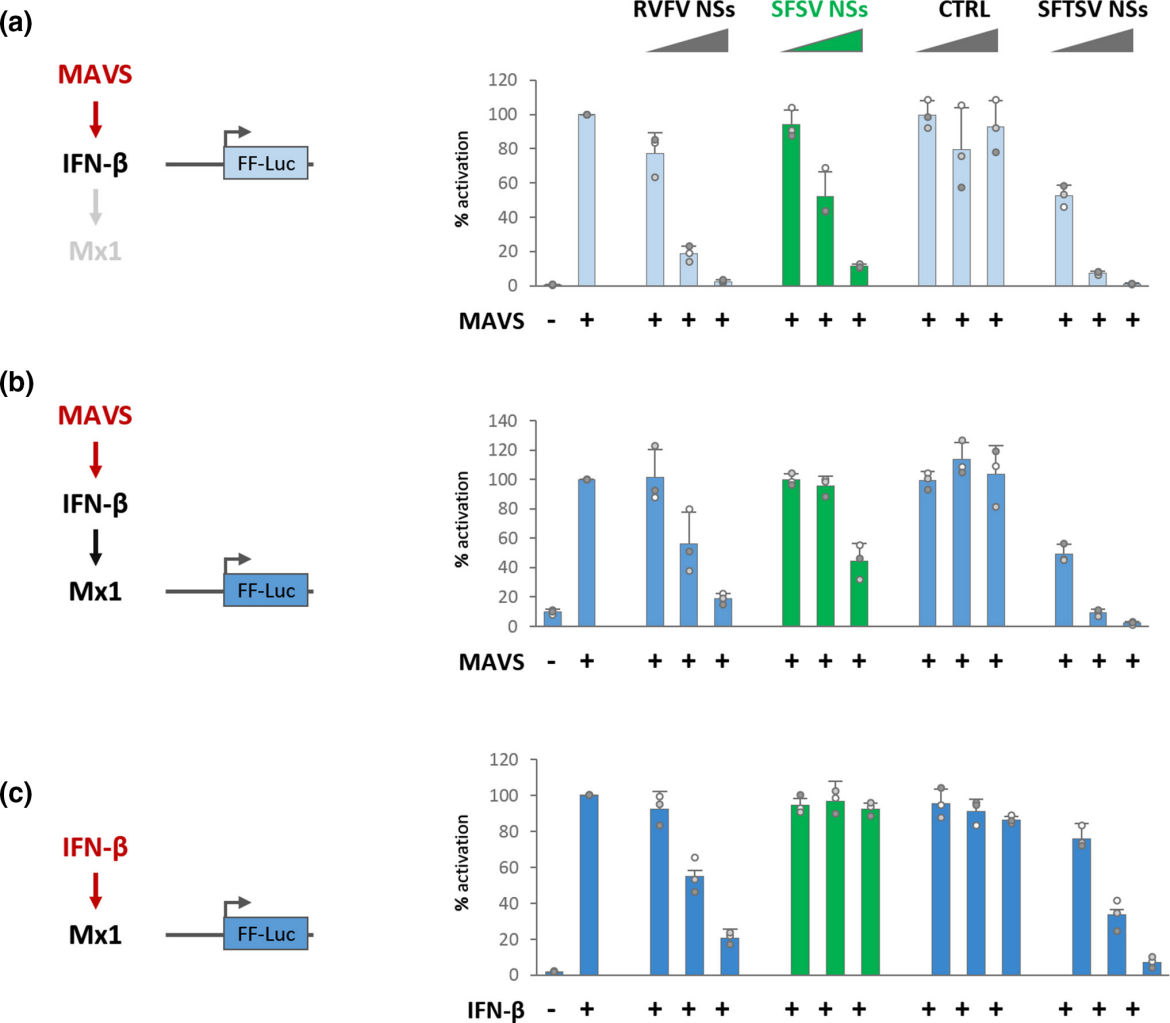
IFN signalling and ISG induction under ectopic SFSV NSs expression. (**a–c)** HEK293 cells were co-transfected with indicated reporter constructs and increasing doses (0.1 ng, 1.0 ng, 10.0 ng) of expression plasmids for 3×FLAG-tagged NSs or negative control (CTRL). IFN induction or signalling was stimulated by concomitant overexpression of MAVS (**a, b**) or addition of 100 IU ml^−1^ IFN-β 12 h after transfection (**c**). Lysates were interrogated for IFN induction (*IFNB1* promoter, **a**) or IFN-dependent ISG induction (*Mx1* promoter, **b, c**) 24 h after stimulation (*n*=3, mean±SD). The respective stimulant is coloured in red.

To dissect the impact of SFSV NSs on IFN induction vs. IFN signalling, we tested ectopically expressed SFSV NSs in luciferase reporter assays. Different promoters and inducers were combined in three experimental set-ups to distinguish the ability of SFSV NSs to block (i) *Ifnb1* promoter induction (Fig. 2a), (ii) indirect *Mx1* promoter stimulation by induced IFN (Fig. 2b), or (iii) direct *Mx1* promoter activation (Fig. 2c). For *Ifnb1* and indirect *Mx1* promoter stimulation, we concomitantly overexpressed the RIG-I adaptor MAVS, as described before [47], whereas direct ISG induction was stimulated by the addition of IFN-β to the medium. Along with SFSV NSs and RVFV NSs, we employed the NSs of the related severe fever with thrombocytopenia syndrome virus (SFTSV, recently reclassified as Dabie bandavirus [57]) as a well-established specific antagonist of both IFN induction and signalling [58–62]. As expected, ectopic SFSV NSs was able to efficiently inhibit *Ifnb1* promoter induction by MAVS (Fig. 2a). In contrast, when the reporter under control of the *Mx1* promoter was employed to measure ISG stimulation by MAVS (i.e. indirectly via secreted IFN), SFSV NSs was less efficient and only inhibitory when given at the highest dose (Fig. 2b). Finally, SFSV NSs completely failed to interfere when *Mx1* promoter activity was stimulated with ectopic IFN-β (Fig. 2c) or IFN-α (data not shown). Thus, SFSV NSs can affect IFN induction, but not IFN signalling.

### SFSV NSs also modulates type III IFN induction

**Fig. 3.**
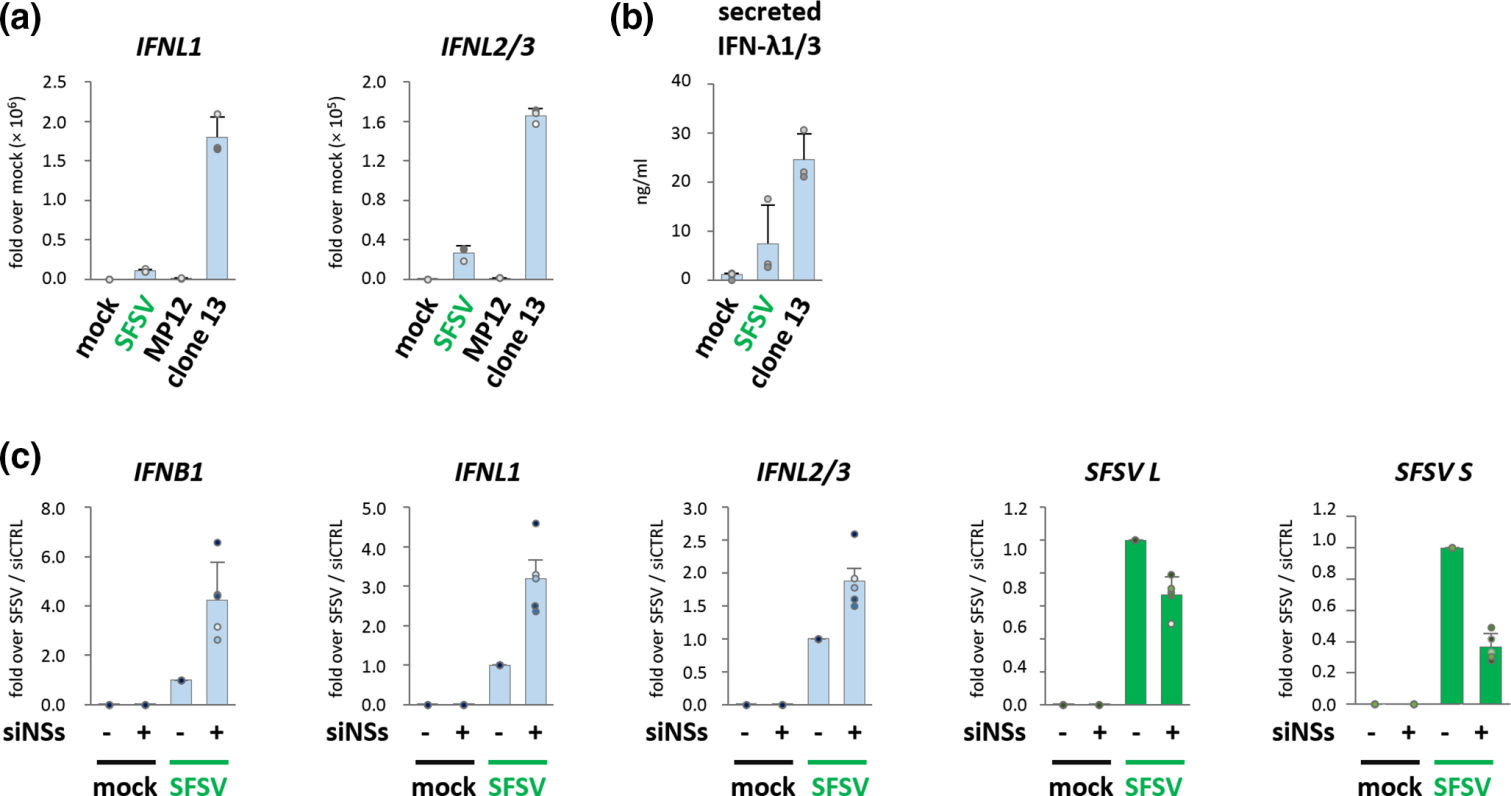
Type III IFN expression under SFSV infection. Samples from Fig. 1b (MOI 1, 12 hpi) were interrogated for type III IFN expression by RT-qPCR (**a**) and supernatants for secreted type III IFNs by ELISA (**b**) (*n*=3, mean±SD). (**c)** A549 cells were subjected to reverse transfection with control siRNA or an siRNA pool targeting SFSV NSs. After 24 h, cells were infected with SFSV (MOI 1) for 12 h and subsequently analysed by RT-qPCR for type I and III IFN, as well as viral L segment and NSs-encoding viral S segment. Control siRNA-treated, SFSV-infected cells were set to 1 (*n*=5, mean±SD).

We found that also the type III IFNs *IFNL1* and *IFNL2/3* were moderately induced by SFSV in A549 cells on the transcriptional level, and low amounts of secreted IFN-λ1 and -λ3 could be detected in cell culture supernatants (Fig. 3a). Moreover, siRNA experiments revealed that the suppression of IFN induction was mediated by NSs (Fig. 3b). Thus, the ISG expression in response to SFSV is most likely due to active infection and reflects a failure of SFSV NSs to fully abrogate type I and III IFN production.

### SFSV NSs fails to sufficiently control IRF-mediated IFN induction

**Fig. 4.**
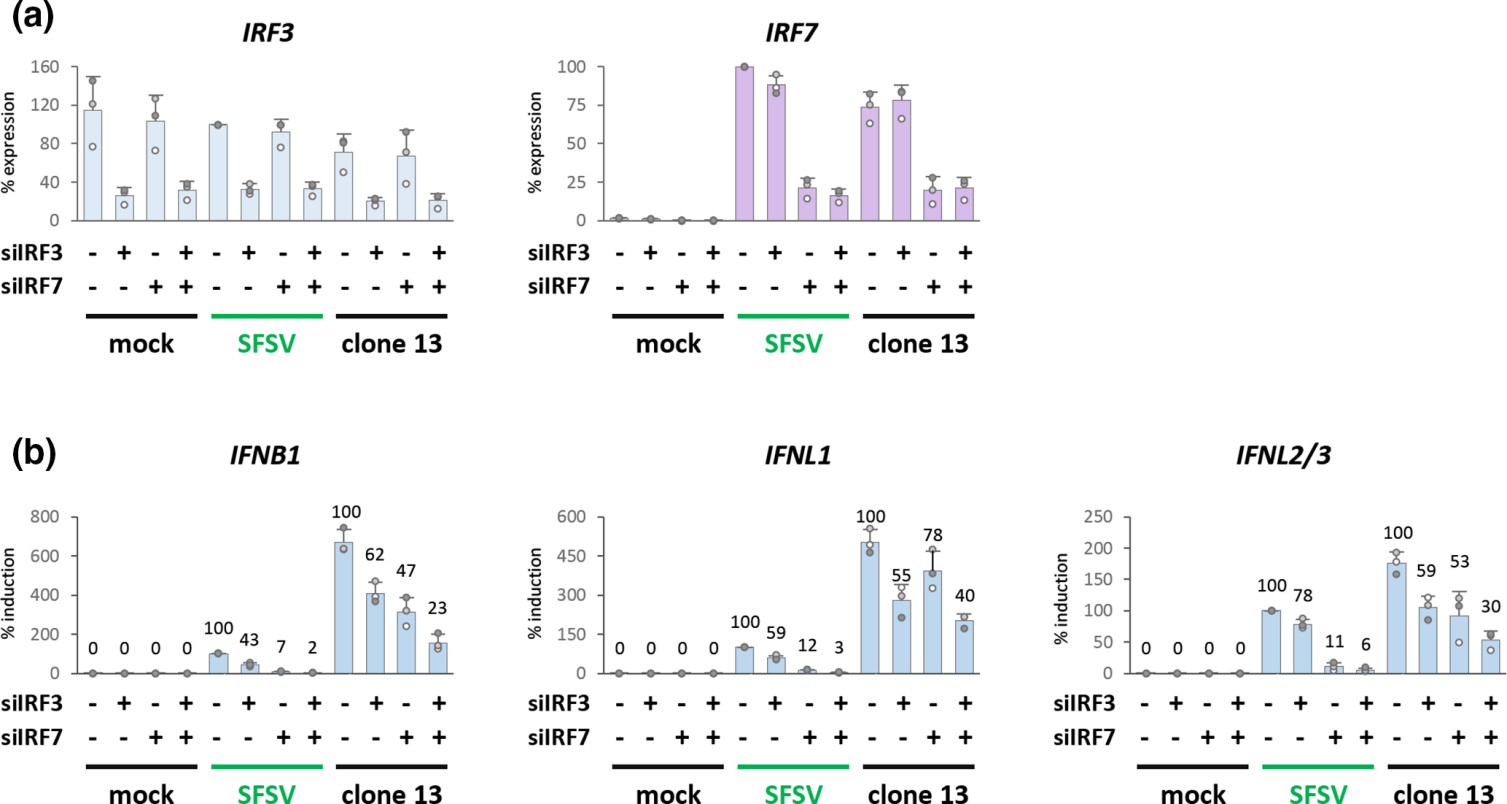
IFN and ISG induction under IRF knockdown. A549 cells were subjected to reverse transfection with control siRNA or siRNA pools targeting IRF3 and IRF7 alone or in combination. After 24 h, cells were infected with SFSV (MOI 1) for 12 h and subsequently analysed for transcript levels of (**a)** IRF3 and IRF7 and (**b)** type I and III IFN (*n*=3, mean±SD). Expression and induction levels were normalized to cells treated with control siRNA and infected with SFSV, and the latter set to 100 %. Data labels represent the percent of IFN induction normalized to the control siRNA condition of the respective virus group.

SFSV NSs might be only a weak IFN induction antagonist due to incomplete sequestration of the cellular IRF3 pool, its inability to target IRF7 [47], or a combination thereof. To test these possibilities, we knocked down either IRF3, IRF7, or both with specific siRNA pools prior to SFSV and clone 13 infection (Fig. 4a). The knockdown of *IRF3* alone partially decreased *IFNB1*, *IFNL1*, and *IFNL2/3* mRNA levels in both clone 13- and SFSV-infected cells (Fig. 4b), suggesting that IRF3 participated in IFN induction in response to SFSV. The knockdown of *IRF7* alone resulted in an even stronger reduction of IFN transcripts in the case of SFSV, whereas for clone 13 the effect was comparable to the one of the *IRF3*-targeting siRNA. This implies that IFN induction under SFSV relied more on IRF7. Finally, the simultaneous knockdown of both IRFs had an additive effect for both viruses, but again for SFSV it led to a stronger reduction of IFN transcripts. Thus, both IRF3 and IRF7 appeared to be responsible for IFN type I and III induction during SFSV infection.

### ISG expression in infected cells depends on IFN signaling

**Fig. 5.**
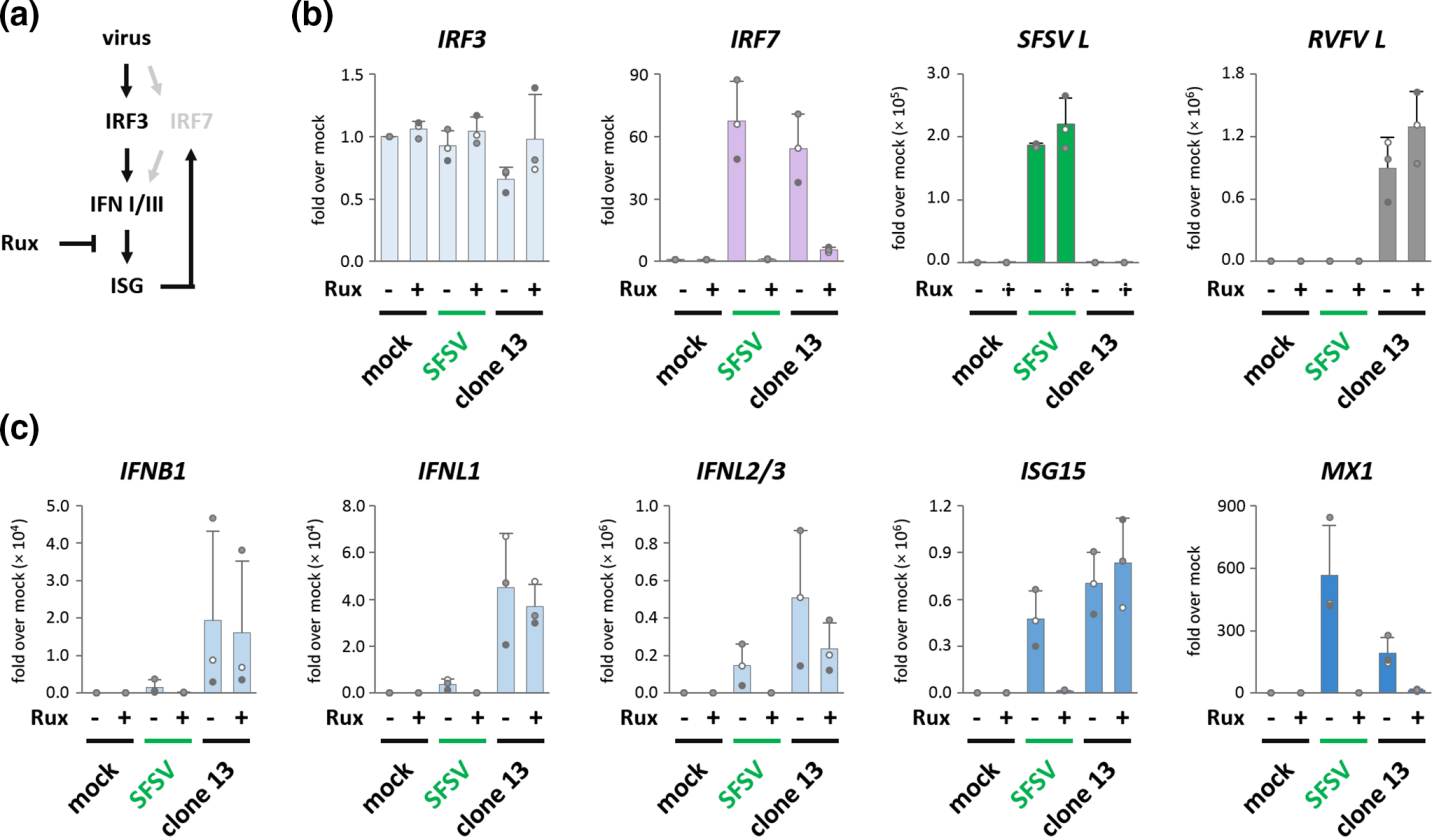
IFN and ISG induction under ruxolitinib treatment. (**a)** In steady-state, type I and III IFN induction relies on IRF3 in most cell types. Upon IFN signalling, however, IRF7 transcription and translation are induced. IRF7 then is activated alongside IRF3 and participates in a positive feedback loop that results in the amplification and diversification of the IFN response. IFN signalling and ISG induction can be abrogated by JAK1/JAK2 inhibitor ruxolitinib (Rux). (**b, c)** A549 cells treated with ruxolitinib or vehicle control from 1 h prior and throughout the infection (MOI 1) until harvest 12 hpi. Cellular RNA was subsequently analysed for transcript levels of IRF3 and IRF7, viral gene segment L, type I and III IFN, as well as ISGs (*n*=3, mean±SD). All samples were normalized to vehicle-treated mock cells.

While IRF3 is constitutively expressed and not regulated by IFN, IRF7 is an ISG itself with only low basic levels in most cell types. Upon IFN signalling, IRF7 is rapidly upregulated on the transcriptional and translational levels, activated along with IRF3, and thereby amplifies IFN induction [28, 63–65] (Fig. 5a). To further differentiate between basally expressed and IFN-induced IRF7, we compared IRF, IFN, and ISG induction under treatment with the JAK1/2 inhibitor ruxolitinib that blocks signalling by IFNs and other cytokines [66]. As expected, ruxolitinib left *IRF3* levels unaffected but blocked the upregulation of *IRF7* (Fig. 5b) and *MX1* (Fig. 5c and data not shown). Similarly, ruxolitinib further decreased the already low inductions of *IFNB1*, *IFNL1*, and *IFNL2/3* in SFSV-infected cells, whereas it had no or only a partially reducing effect on the IFN transcripts in clone 13-infected cells. Finally, *ISG15* expression remained at baseline level under simultaneous ruxolitinib treatment and SFSV infection, whereas it was readily induced by clone 13 also when JAK-dependent signalling was inhibited. Hence, the IRF7-mediated positive feedback loop via secreted IFNs and perhaps other cytokines seems to be critical for the IFN and ISG inductions that are observed in SFSV-infected cells.

## Discussion

Phleboviruses cover a wide spectrum of virulence. As the majority of novel phleboviruses are currently identified by screening of putative arthropod vectors, their potential to cause disease in humans is mostly unknown.

The importance of the IFN system in the outcome of phlebovirus infection has been illustrated in animal models of infection by (a) the increased susceptibility of IFN-deficient mice, (b) the protective effect of prophylactic and early therapeutic application of type I IFNs, and (c) the association of an early type I IFN response with survival (see introduction). A major part of the antiviral activity is thereby mediated by ISGs acting on at multiple levels of the viral replication cycle [28]. To date, a systematic analysis of ISGs for anti-phleboviral activity is lacking. Nevertheless, a small set of ISGs has been shown to restrict the replication of RVFV [3, 43]. Similar to RVFV, SFSV is restricted by both overexpression of MxA and ectopic type I IFN if present during early stages of the viral replication cycle [30, 33]. Accordingly, phleboviruses have evolved a number of strategies to counteract IFN induction. Well established examples are the NSs proteins of virulent RVFV and TOSV that promote proteasomal degradation of host factors, either by recruiting the host ubiquitination machinery to target proteins or by NSs acting as ubiquitin ligase itself, respectively [45, 48]. In contrast, NSs of the only mildly virulent SFSV acts by stoichiometric interaction with IRF-3. In a similar manner, NSs of the related, highly virulent SFTSV bandavirus sequesters multiple factors of the IFN induction pathway into inclusion bodies, whereas NSs of the apathogenic Uukuniemi virus (UUKV) is a weak IFN antagonist [59–62, 67]. It has been discussed that the IFN-antagonistic activity of an NSs protein may correlate with the virulence of the respective phlebovirus [3, 43, 62], and that novel phleboviruses (or their NSs proteins) are more habitually tested for inhibition of IFN induction. Interestingly, NSs proteins of the virulent tick-borne bandaviruses also specifically target IFN signalling, whereas UUKV NSs does not [62]. This may suggest that, besides differences in breadth and speed of host factor inactivation, antagonism of both IFN induction and IFN signalling is required for high virulence. To our knowledge, such a comparative analysis has not been reported yet for phleboviruses - probably due to the fact that RVFV NSs blunts host gene expression and is therefore expected to abrogate IFN signalling and ISG induction. Here, we thus characterized the antagonistic capacity of mildly pathogenic SFSV towards IFN signalling and ISG induction in comparison with RVFV.

As reported previously for *IFNB1* [47], the induction of type III IFNs was dampened but not abrogated by SFSV NSs. IFN signalling was clearly activated in response to SFSV infection, and ISGs were induced on both the transcript and protein levels. IFN signalling upon addition of ectopic IFN did not increase ISG expression further and was inhibited neither by SFSV infection nor by NSs overexpression, in agreement with the observation that NSs did not interact with STAT1 or STAT2 (data not shown) and the absence of any IFN signalling factors from the NSs interactome [68, 69]. Interestingly, IFN and subsequent ISG induction were driven predominantly by IRF7, consistent with the failure of SFSV NSs to target IRF7 in our previous study [68]. In addition to IRF7, a role for IRF5 has been implied in IFN induction and a mouse model for Oropouche and LaCrosse orthobunyavirus infection [70]. Similar to IRF7, IRF5 is not targeted by SFSV NSs [47]. However, unlike what we observed for IRF7, knockdown of *IRF5* did not lead to any reduction in IFN or ISG levels in our experimental system (data not shown). In summary, the IFN-antagonistic activity of SFSV NSs is limited to and thereby relying entirely on its ability to modulate IRF3-driven *IFNB1* induction. Given that NSs needs to be produced freshly in infected cells and that already incoming viral genome segments can activate innate sensing [25], SFSV NSs appears to be a rather weak and inefficient IFN antagonist. All taken together, we propose the following model for SFSV infection: although type I and III IFN induction are down-modulated by NSs-mediated IRF3 sequestration, they cannot be sufficiently abrogated due to an (initial) excess of IRF3 over newly generated NSs. Secretion of small amounts of first-wave IFN-β and IFN-λ then triggers IFN signalling, unhindered by NSs, and the transcriptional and translational upregulation of IRF7. The latter, again unaffected by SFSV NSs, further amplifies the IFN and ISG response in the infected cells, resulting in substantial ISG induction. Of note, we recently found that SFSV NSs, in order to evade restriction by the powerful ISG product PKR, interacts with the translation initiation factor 2B (eIF2B), resulting in enhanced cap-dependent translation [69]. While ensuring the synthesis of viral proteins, this probably also augments the production of IFNs and ISGs within infected cells. Thereby, IFN from SFSV-infected cells can not only establish an antiviral state in bystander cells, but also in cells already infected.

Unfortunately, no small animal model is available to study SFSV infection [12]. However, early reports firmly established the self-limited nature of the febrile disease caused by SFSV in men [11, 12] and it is conceivable that, when spreading in a mammalian organism, the virus quickly encounters cells with high IRF levels. Accordingly, while targeting IRF3 might allow the virus a head start, considerable IFN induction through the IRF7-dependent positive feedback loop and possibly also by professional cell types with intrinsically high IRF7 levels can quickly limit viral spread. Hence, it is tempting to speculate that the failure of SFSV NSs to sufficiently blunt IFN induction and to affect IFN signalling significantly contributes to its limited virulence in mammalian hosts. While additional factors such as polymerase efficiency and receptor tropism, both of which remain unexplored in the case of SFSV, are contributing to virulence, studies using recombinant chimeric phleboviruses support a lead role of SFSV NSs in virulence: when replacing RVFV NSs with other NSs genes, the substitution with SFSV NSs conferred substantial attenuation in the mouse model and the chimeric virus has even been suggested as vaccine candidate for RVFV [46, 71].

Together with the mentioned studies using the phlebo-like bandaviruses in cells or chimeric phleboviruses in the mouse model, our data now provides further evidence that the ability of a particular NSs to interfere with both IFN induction and signalling are required for high virulence. Therefore, we propose that, rather than testing only for IFN induction, both IFN induction and signalling should be taken into considerationwhen rating the potential virulence of a novel phlebo- or bandavirus.

## Supplementary Data

Supplementary material 1

## References

[1] Alkan C, Bichaud L, de Lamballerie X, Alten B, Gould EA (2013). Sandfly-borne phleboviruses of Eurasia and Africa: epidemiology, genetic diversity, geographic range, control measures. Antiviral Res.

[2] Elliott RM, Brennan B (2014). Emerging phleboviruses. Curr Opin Virol.

[3] Wuerth JD, Weber F (2016). Phleboviruses and the type I interferon response. Viruses.

[4] Carhan A, Uyar Y, Ozkaya E, Ertek M, Dobler G (2010). Characterization of a sandfly fever Sicilian virus isolated during a sandfly fever epidemic in Turkey. J Clin Virol.

[5] Ergunay K, Ayhan N, Charrel RN (2017). Novel and emergent sandfly-borne phleboviruses in Asia Minor: A systematic review. Rev Med Virol.

[6] Ayhan N, Velo E, de Lamballerie X, Kota M, Kadriaj P (2016). Detection of Leishmania infantum and a novel phlebovirus (balkan virus) from sand flies in Albania. Vector Borne Zoonotic Dis.

[7] Marklewitz M, Dutari LC, Paraskevopoulou S, Page RA, Loaiza JR (2019). Diverse novel phleboviruses in sandflies from the Panama Canal area, central Panama. J Gen Virol.

[8] Marklewitz M, Tchouassi DP, Hieke C, Heyde V, Torto B (2020). Insights into the evolutionary origin of Mediterranean sandfly fever viruses. mSphere.

[9] Ohlendorf V, Marklewitz M, Kopp A, Yordanov S, Drosten C (2019). Huge diversity of phleboviruses in ticks from Strandja Nature Park, Bulgaria. Ticks Tick Borne Dis.

[10] Papa A, Velo E, Bino S (2011). A novel phlebovirus in *Albanian sandflies*. Clin Microbiol Infect.

[11] Bartelloni PJ, Tesh RB (1976). Clinical and serologic responses of volunteers infected with phlebotomus fever virus (Sicilian type. Am J Trop Med Hyg.

[12] Sabin AB (1951). Experimental studies on Phlebotomus (pappataci, sandfly) fever during World War II. Arch Gesamte Virusforsch.

[13] Alwassouf S, Christodoulou V, Bichaud L, Ntais P, Mazeris A (2016). Seroprevalence of sandfly-borne phleboviruses belonging to three serocomplexes (Sandfly fever Naples, Sandfly fever Sicilian and Salehabad) in dogs from Greece and cyprus using neutralization test. PLoS Negl Trop Dis.

[14] Ayhan N, Sherifi K, Taraku A, Berxholi K, Charrel RN (2017). High rates of neutralizing antibodies to toscana and sandfly fever sicilian viruses in Livestock, Kosovo. Emerg Infect Dis.

[15] Eitrem R, Stylianou M, Niklasson B (1991). High prevalence rates of antibody to three sandfly fever viruses (Sicilian, Naples, and Toscana) among Cypriots. Epidemiol Infect.

[16] Sakhria S, Alwassouf S, Fares W, Bichaud L, Dachraoui K (2014). Presence of sandfly-borne phleboviruses of two antigenic complexes (Sandfly fever Naples virus and Sandfly fever Sicilian virus) in two different bio-geographical regions of Tunisia demonstrated by a microneutralisation-based seroprevalence study in dogs. Parasit Vectors.

[17] Tesh RB, Saidi S, Gajdamovic SJ, Rodhain F, Vesenjak-Hirjan J (1976). Serological studies on the epidemiology of sandfly fever in the Old World. Bull World Health Organ.

[18] Eitrem R, Vene S, Niklasson B (1990). Incidence of sand fly fever among Swedish United Nations soldiers on Cyprus during 1985. Am J Trop Med Hyg.

[19] Eitrem R, Niklasson B, Weiland O (1991). Sandfly fever among Swedish tourists. Scand J Infect Dis.

[20] Kniha E, Obwaller AG, Dobler G, Poeppl W, Mooseder G (2019). Phlebovirus seroprevalence in Austrian Army personnel returning from missions abroad. Parasit Vectors.

[21] Papa A, Konstantinou G, Pavlidou V, Antoniadis A (2006). Sandfly fever virus outbreak in Cyprus. Clin Microbiol Infect.

[22] Shiraly R, Khosravi A, Farahangiz S (2017). Seroprevalence of sandfly fever virus infection in military personnel on the western border of Iran. J Infect Public Health.

[23] Alkan C, Erisoz Kasap O, Alten B, Lamballerie de, Charrel RN (2016). Sandfly-Borne Phlebovirus Isolations from Turkey: New Insight into the Sandfly fever Sicilian and Sandfly fever Naples Species. PLoS Negl Trop Dis.

[24] Alkan C, Moin Vaziri V, Ayhan N, Badakhshan M, Bichaud L (2017). Isolation and sequencing of Dashli virus, a novel sicilian-like virus in sandflies from Iran; Genetic and phylogenetic evidence for the creation of one novel species within the phlebovirus genus in the Phenuiviridae family. PLoS Negl Trop Dis.

[25] Weber M, Gawanbacht A, Habjan M, Rang A, Borner C (2013). Incoming RNA virus nucleocapsids containing a 5’-triphosphorylated genome activate RIG-I and antiviral signaling. Cell Host Microbe.

[26] Liu G, Gack MU (2020). Distinct and orchestrated functions of RNA sensors in innate immunity. Immunity.

[27] Yoneyama M, Onomoto K, Jogi M, Akaboshi T, Fujita T (2015). Viral RNA detection by RIG-I-like receptors. Curr Opin Immunol.

[28] Schoggins JW (2019). Interferon-stimulated genes: what do they all do?. Annu Rev Virol.

[29] do Valle TZ, Billecocq A, Guillemot L, Alberts R, Gommet C (2010). A new mouse model reveals a critical role for host innate immunity in resistance to Rift Valley fever. J Immunol.

[30] Frese M, Kochs G, Feldmann H, Hertkorn C, Haller O (1996). Inhibition of bunyaviruses, phleboviruses, and hantaviruses by human MxA protein. J Virol.

[31] Sandrock M, Frese M, Haller O, Kochs G (2001). Interferon-induced rat Mx proteins confer resistance to Rift Valley fever virus and other arthropod-borne viruses. J Interferon Cytokine Res.

[32] Bouloy M, Janzen C, Vialat P, Khun H, Pavlovic J (2001). Genetic evidence for an interferon-antagonistic function of Rift Valley fever virus nonstructural protein NSs. J Virol.

[33] Crance JM, Gratier D, Guimet J, Jouan A (1997). Inhibition of sandfly fever Sicilian virus (Phlebovirus) replication in vitro by antiviral compounds. Res Virol.

[34] Habjan M, Pichlmair A, Elliott RM, Overby AK, Glatter T (2009). NSs protein of Rift Valley fever virus induces the specific degradation of the double-stranded RNA-dependent protein kinase. J Virol.

[35] Kende M (1985). Prophylactic and therapeutic efficacy of poly(I,C)-LC against Rift Valley fever virus infection in mice. J Biol Response Mod.

[36] Liu Y, Wu B, Paessler S, Walker DH, Tesh RB (2014). The pathogenesis of severe fever with thrombocytopenia syndrome virus infection in alpha/beta interferon knockout mice: insights into the pathologic mechanisms of a new viral hemorrhagic fever. J Virol.

[37] Mendenhall M, Wong MH, Skirpstunas R, Morrey JD, Gowen BB (2009). Punta Toro virus (Bunyaviridae, Phlebovirus) infection in mice: strain differences in pathogenesis and host interferon response. Virology.

[38] Morrill JC, Jennings GB, Cosgriff TM, Gibbs PH, Peters CJ (1989). Prevention of Rift Valley fever in rhesus monkeys with interferon-alpha. Rev Infect Dis.

[39] Morrill JC, Jennings GB, Johnson AJ, Cosgriff TM, Gibbs PH (1990). Pathogenesis of Rift Valley fever in rhesus monkeys: role of interferon response. Arch Virol.

[40] Peters CJ, Reynolds JA, Slone TW, Jones DE, Stephen EL (1986). Prophylaxis of Rift Valley fever with antiviral drugs, immune serum, an interferon inducer, and a macrophage activator. Antiviral Res.

[41] Sidwell RW, Huffman JH, Smee DF, Gilbert J, Gessaman A (1992). Potential role of immunomodulators for treatment of phlebovirus infections of animals. Ann N Y Acad Sci.

[42] Eifan S, Schnettler E, Dietrich I, Kohl A, Blomstrom AL (2013). Non-structural proteins of arthropod-borne bunyaviruses: roles and functions. Viruses.

[43] Ly HJ, Ikegami T (2016). Rift Valley fever virus NSs protein functions and the similarity to other bunyavirus NSs proteins. Virol J.

[44] Ikegami T, Narayanan K, Won S, Kamitani W, Peters CJ (2009). Rift Valley fever virus NSs protein promotes post-transcriptional downregulation of protein kinase PKR and inhibits eIF2alpha phosphorylation. PLoS Pathog.

[45] Kainulainen M, Habjan M, Hubel P, Busch L, Lau S (2014). Virulence factor NSs of rift valley fever virus recruits the F-box protein FBXO3 to degrade subunit p62 of general transcription factor TFIIH. J Virol.

[46] Lihoradova OA, Indran SV, Kalveram B, Lokugamage N, Head JA (2013). Characterization of Rift Valley fever virus MP-12 strain encoding NSs of Punta Toro virus or sandfly fever Sicilian virus. PLoS Negl Trop Dis.

[47] Wuerth JD, Habjan M, Wulle J, Superti-Furga G, Pichlmair A (2018). NSs Protein of Sandfly Fever Sicilian Phlebovirus Counteracts Interferon (IFN) Induction by Masking the DNA-Binding Domain of IFN Regulatory Factor 3. J Virol.

[48] Gori Savellini G, Anichini G, Gandolfo C, Prathyumnan S, Cusi MG (2019). Toscana virus non-structural protein NSS acts as E3 ubiquitin ligase promoting rig-i degradation. PLoS Pathog.

[49] Horisberger MA, de Staritzky K (1987). A recombinant human interferon-alpha B/D hybrid with a broad host-range. J Gen Virol.

[50] Yoneyama M, Suhara W, Fukuhara Y, Fukuda M, Nishida E (1998). Direct triggering of the type I interferon system by virus infection: activation of a transcription factor complex containing IRF-3 and CBP/p300. EMBO J.

[51] Kochs G, Garcia-Sastre A, Martinez-Sobrido L (2007). Multiple anti-interferon actions of the influenza A virus NS1 protein. J Virol.

[52] Weidmann M, Sanchez-Seco MP, Sall AA, Ly PO, Thiongane Y (2008). Rapid detection of important human pathogenic Phleboviruses. J Clin Virol.

[53] Bird BH, Bawiec DA, Ksiazek TG, Shoemaker TR, Nichol ST (2007). Highly sensitive and broadly reactive quantitative reverse transcription-PCR assay for high-throughput detection of Rift Valley fever virus. J Clin Microbiol.

[54] Holzer M, Schoen A, Wulle J, Muller MA, Drosten C (2019). Virus- and interferon alpha-induced transcriptomes of cells from the microbat *Myotis daubentonii*. iScience.

[55] Prescott J, Hall P, Acuna-Retamar M, Ye C, Wathelet MG (2010). New World hantaviruses activate IFNlambda production in type I IFN-deficient vero E6 cells. PLoS One.

[56] Kuri T, Habjan M, Penski N, Weber F (2010). Species-independent bioassay for sensitive quantification of antiviral type I interferons. Virol J.

[57] International Committee on Taxonomy of Viruses (2019). Virus Taxonomy: 2019 Release. https://talk.ictvonline.org/taxonomy/.

[58] Chaudhary V, Zhang S, Yuen KS, Li C, Lui PY (2015). Suppression of type I and type III IFN signalling by NSs protein of severe fever with thrombocytopenia syndrome virus through inhibition of STAT1 phosphorylation and activation. J Gen Virol.

[59] Ning YJ, Wang M, Deng M, Shen S, Liu W (2014). Viral suppression of innate immunity via spatial isolation of TBK1/IKKepsilon from mitochondrial antiviral platform. J Mol Cell Biol.

[60] Qu B, Qi X, Wu X, Liang M, Li C (2012). Suppression of the interferon and NF-kappaB responses by severe fever with thrombocytopenia syndrome virus. J Virol.

[61] Santiago FW, Covaleda LM, Sanchez-Aparicio MT, Silvas JA, Diaz-Vizarreta AC (2014). Hijacking of RIG-I signaling proteins into virus-induced cytoplasmic structures correlates with the inhibition of type I interferon responses. J Virol.

[62] Rezelj VV, Li P, Chaudhary V, Elliott RM, Jin DY (2017). Differential antagonism of human innate immune responses by tick-borne phlebovirus nonstructural proteins. mSphere.

[63] Ning S, Pagano JS, Barber GN (2011). IRF7: activation, regulation, modification and function. Genes Immun.

[64] Marie I, Durbin JE, Levy DE (1998). Differential viral induction of distinct interferon-alpha genes by positive feedback through interferon regulatory factor-7. EMBO J.

[65] Sato M, Hata N, Asagiri M, Nakaya T, Taniguchi T (1998). Positive feedback regulation of type I IFN genes by the IFN-inducible transcription factor IRF-7. FEBS Lett.

[66] Stewart CE, Randall RE, Adamson CS (2014). Inhibitors of the interferon response enhance virus replication in vitro. PLOS ONE.

[67] Wu X, Qi X, Qu B, Zhang Z, Liang M (2014). Evasion of antiviral immunity through sequestering of TBK1/IKKepsilon/IRF3 into viral inclusion bodies. J Virol.

[68] Pichlmair A, Kandasamy K, Alvisi G, Mulhern O, Sacco R (2012). Viral immune modulators perturb the human molecular network by common and unique strategies. Nature.

[69] Wuerth JD, Habjan M, Kainulainen M, Berisha B, Bertheloot D (2020). eIF2B as a target for viral evasion of PKR-Mediated Translation Inhibition. mBio.

[70] Proenca-Modena JL, Hyde JL, Sesti-Costa R, Lucas T, Pinto AK (2016). Interferon-Regulatory Factor 5-dependent signaling restricts orthobunyavirus dissemination to the central nervous System. J Virol.

[71] Nishiyama S, Slack OA, Lokugamage N, Hill TE, Juelich TL (2016). Attenuation of pathogenic Rift Valley fever virus strain through the chimeric S-segment encoding sandfly fever phlebovirus NSs or a dominant-negative PKR. Virulence.

