# Supplementary material for: NSs of the mildly virulent sandfly fever Sicilian virus is unable to inhibit interferon signaling and upregulation of interferon-stimulated genes
Source: J Gen Virol. 2021 Nov 2;102(11):001676. doi: 10.1099/jgv.0.001676 (PMC8742993; doi:10.1099/jgv.0.001676)
Supplement: Supplementary material 1 [file jgv-102-1676-s001.pdf]

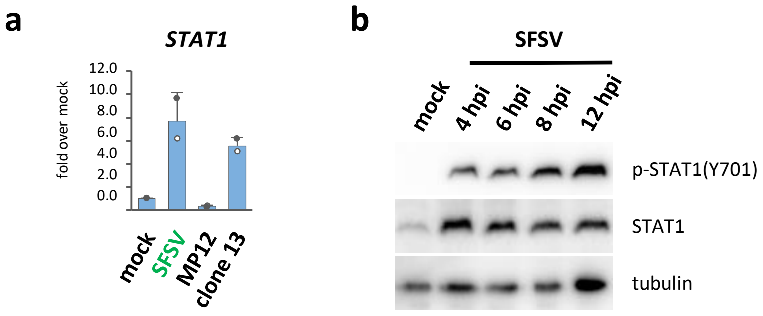

Supporting Fig. 1: STAT1 expression and phosphorylation under infection

a Samples of Fig. 1b were analysed via RT-qPCR with primers for *STAT1* (n = 2, mean ± SD). **b** A549 cells were infected with SFSV (MOI 1), harvested at the indicated time points, and analysed by immunoblotting for STAT1 phosphorylation and total STAT1 levels as described for Fig. 1a.

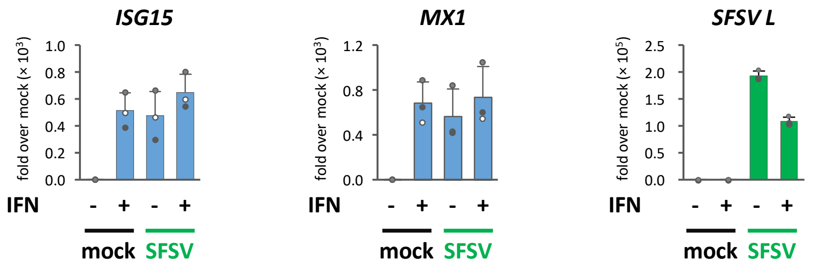

Supporting Fig. 2: ISG induction under IFN pre-treatment
A549 cells were pre-treated with 100 IU/ml pan-species IFN-α (B/D) for 1 h, subsequently infected with SFSV (MOI 1) or mock-treated, and IFN or mock treatment was continued until harvesting for RT-qPCR analysis 12 hpi (n = 3, mean ± SD).

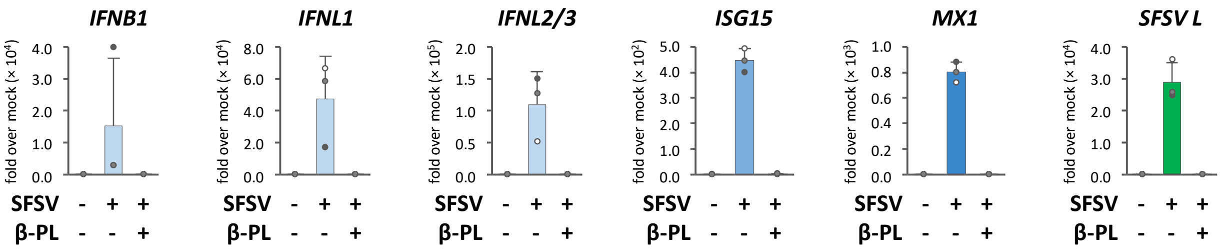

Supporting Fig. 3: Interferon and ISG induction after virus inactivation

A549 cells were infected with β-propiolactone-inactivated or matching mock-treated SFSV (MOI 1), and harvested 12 hpi for RT-qPCR analysis (n = 3, mean ± SD).
